# Short- and long-term survival after out-of-hospital cardiac arrest in Kaunas (Lithuania) from 2016 to 2018

**DOI:** 10.1186/s12872-022-02964-4

**Published:** 2022-12-03

**Authors:** Linas Darginavicius, Ilona Kajokaite, Nerijus Mikelionis, Jone Vencloviene, Paulius Dobozinskas, Egle Vaitkaitiene, Dinas Vaitkaitis, Asta Krikscionaitiene

**Affiliations:** 1 Department of Disaster Medicine, Lithuanian University of Health Sciences, Eiveniu 4-512, 50161 Kaunas, Lithuania; 2 Kaunas Emergency Medical Service Station, Kaunas, Lithuania; 3 Department of Environmental Sciences, Faculty of Natural Sciences, Vytautas Magnus University, Kaunas, Lithuania; 4 Department of Public Health, Lithuanian University of Health Sciences, Kaunas, Lithuania

**Keywords:** Out-of-hospital cardiac arrest, Utstein, Incidence, Emergency medical services, Survival, Outcomes, Prehospital resuscitation

## Abstract

**Background:**

No studies analysing out-of-hospital cardiac arrest (OHCA) epidemiology and outcomes in Lithuania were published in the last decade.

**Methods:**

We conducted a retrospective analysis of prospectively collected data. The incidence of OHCA and the demographics and outcomes of patients who were treated for OHCA between 1 and 2016 and 31 December 2018 at Kaunas Emergency Medical Service (EMS) were collected and are reported in accordance with the Utstein recommendations. Multivariable logistic regression analysis was used to identify predictors of survival to hospital discharge.

**Results:**

In total, 838 OHCA cases of EMS-treated cardiac arrest (CA) were reported (95.8 per 100.000 inhabitants). The median age was 71 (IQR 58–81) years of age, and 66.7% of patients were males. A total of 73.8% of OHCA cases occurred at home, 59.3% were witnessed by a bystander, and 54.5% received bystander cardiopulmonary resuscitation. The median EMS response time was 10 min. Cardiac aetiology was the leading cause of CA (78.8%). The initial rhythm was shockable in 27.6% of all cases. Return of spontaneous circulation at hospital transfer was evident in 24.9% of all cases. The survival to hospital discharge rate was 10.9%, and the 1-year survival rate was 6.9%. The survival to hospital discharge rate in the Utstein comparator group was 36.1%, and the 1-year survival rate was 27.2%. Five factors were associated with improved survival to hospital discharge: shockable rhythm, time from call to arrival at the patient less than 10 min, witnessed OHCA, age < 80 years, and male sex.

**Conclusion:**

This is the first OHCA study from Lithuania examining OHCA epidemiology and outcomes over a three year period. Routine OHCA data collection and analysis will allow us to track the efficacy of service improvements and should become a standard practice in all Lithuanian regions.

*Trial registration*: This research was registered in the clinicaltrials.gov database: Identifiers: NCT04784117, Unique Protocol ID: LITOHCA. Brief Title: Out-of-hospital Cardiac Arrest Epidemiology and Outcomes in Kaunas 2016–2021.

## Background

While the global survival rate of out-of-hospital cardiac arrest (OHCA) has significantly improved in the past 40 years [1], it remains poor. The overall survival to hospital discharge rate is less than 10% [1, 2]. The EuReCa TWO study reported outcomes of OHCA from 28 European countries [2]. Unfortunately, there were no data from Lithuania in the EuReCa Two study. In Lithuania, the incidence of OHCA is unknown, as there is no official coding for OHCA as a cause of death in the national death registry.

The epidemiology of OHCA in Kaunas, the second largest city in Lithuania, has not been systematically reported in the last decade. Kaunas Emergency Medical Service (EMS) has undergone some major stepwise changes since 2011, including implementation of the Medical Priority Dispatch System (MPDS) and dispatcher-assisted cardiopulmonary resuscitation (CPR) instructions.

The aim of our study was to explore the epidemiology and outcomes of OHCA in Kaunas and to examine the impact of different patient and care factors on survival to hospital discharge.

## Methods

### Study design

We conducted a retrospective analysis of prospectively collected data from Kaunas EMS-attended OHCA cases in which resuscitation was attempted from 1 to 2016 to 31 December 2018.

### Data sources

We used four data sources to describe each OHCA event: (1) Kaunas EMS Dispatcher Centre data, (2) EMS data, (3) hospital data, and (4) death registry data. EMS dispatcher data and EMS recordings were collected from the Kaunas EMS digital databases. Each OHCA case in which EMS staff initiated CPR underwent an internal audit by a Kaunas EMS quality manager and was included in the study. EMS dispatcher calls were reviewed by the EMS dispatch quality manager. Hospital data were collected from both paper records and the hospital information system (started in June 2017). Hospital data were retrieved manually and collected in the study database. The 1-year survival of patients discharged alive from a hospital was retrieved from the Lithuanian Health Information Centre of Institute of Hygiene, which is responsible for national death statistics in Lithuania.

### Study settings

In 2018, Lithuania had a population of 2,808,901 and occupied an area of 65,300 km2. Approximately 70% of the Lithuanian population lives in cities. Kaunas is the second largest Lithuanian city, with a population of approximately 0.29 million. The Kaunas EMS Station is the only prehospital care provider in the city. The dispatch system is entirely protocol-based. In Kaunas, all the callers were instructed to perform dispatcher-assisted CPR (DA-CPR) using the standard MPDS ProQA® cardiac arrest (CA) protocol starting in 2011. The EMS is a two-tiered response system: a basic life support (BLS) tier with paramedics or a nurse and a paramedic who can apply an automated external defibrillator (AED) and an advanced life support (ALS) tier with ambulance teams including a physician and/or a nurse with advanced competencies in emergency medicine and a paramedic. In the case of presumed OHCA, a dispatcher always dispatches two EMS teams: the one closest to the victim and the ALS team. In Lithuania, CPR regulations are based on an order of the Ministry of Health, which was drafted under the European Resuscitation Council (ERC) guidelines. We do not have a do-not-resuscitate (DNAR) order in Lithuania.

### Patient population

All OHCA cases in which EMS staff initiated CPR were included in the study. OHCA was defined as the cessation of cardiac mechanical activities as confirmed by the absence of signs of circulation [3]. Patients who received bystander CPR but had a pulse when EMS staff arrived were not included in the study, except for one patient who received a shock from an AED before EMS arrival.

The exclusion criteria were age less than 18 years and obvious signs of death on EMS arrival.

### Variables

The core study dataset complied with the Utstein definitions [3] and is presented in Tables 1 and 2. We collected and examined 27 core and supplemental variables: system (population served, number of CAs attended, number of resuscitation attempted and not attempted, system description), dispatcher (dispatcher identified presence of CA, dispatcher provided CPR instructions), patient (age, sex, witnessed arrest, arrest location, bystander response, first monitored rhythm, aetiology), process (response times, defibrillation time, provision of targeted temperature management (TTM), drugs, performance of coronary angiography, number of occluded arteries); outcomes (prehospital ROSC, survived event, survival to hospital discharge, 1-year survival, transport to hospital, neurological outcome at discharge and discharge location).

**Table 1:** Out-of-hospital cardiac arrest patient characteristics

Characteristics	Total/all cases	Utstein group
Number (%)	838 (100)	138 (16.5)
Incidence rate per 100,000 inhabitants per year	95.4	15.7
*Dispatcher identified cardiac arrest*		
Yes	419 (50.0)	81 (58.7)
No	103 (12.3)	11 (8.0)
N/A (was alive)	157 (18.7)	13 (9.4)
Missing	159 (19.0)	33 (23.9)
*Dispatcher CPR*		
Yes	374 (44.6)	73 (52.9)
No	146 (17.4)	19 (13.8)
N/A (was alive)	155 (18.5)	13 (9.4)
Missing	163 (19.5)	33 (23.9)
*Age*		
Median	71 years (IQR, 58–81)	70.5 (IQR 59–77)
Min–max	18–99	33–91
Missing	2 (0.2)	0 (0.0)
*Sex*		
Male	559 (66.7)	112 (81.2)
Female	279 (33.3)	26 (18.8)
*Location*		
Residence	607 (73.8)	88 (63.8)
Nursing home/medical facility	43 (5.2)	6 (4.3)
Public area	173 (21.0)	44 (31.9)
Missing	15 (1.8)	0 (0.0)
*Aetiology witnessed arrest*		
Medical/cardiac cause	660 (78.8)	138 (100)
Bystander	497 (59.3)	138 (100)
EMS	100 (11.9)	
Unwitnessed	103 (12.3)	
Missing	138 (16.5)	
*Bystander CPR*		
Yes	457 (54.5)	109 (79.0)
No	194 (23.2)	23 (16.7)
N/A (was alive)	168 (20.0)	6 (4.3)
Missing	19 (2.3)	0 (0.0)
*Initial cardiac rhythm*		
VF/pVT	231 (27.5)	138 (100)
PEA/asystole	576 (68.5)	
Missing	32 (3.8)	
*EMS defibrillation*		
Yes	231 (27.6)	138 (100)
No	607 (72.4)	
*Epinephrine*		
Yes	760 (90.7)	113 (81.9)
No	78 (9.3)	25 (18.1)
*Transported to hospital*		
Yes	249 (29.7)	78 (56.5)
Specialist centre	216 (86.7)	78 (56.5)
Non-specialist centre	33 (13.3)	0 (0)
*Coronarography attempted*		
Yes	97 (40.8)*	59 (59.0)**
*Number of occluded coronary arteries*		
0	14 (14.7)	8 (13.6)
1	23 (24.2)	16 (27.1)
2	19 (20.0)	13 (22.0)
3	39 (41.1)	22 (37.3)
Missing	2 (2.1)	0 (0)
*Targeted temperature management*		
Performed	5 (2,1)*	1 (1.3)**
Indicated, but not performed	152 (63.9)*	52 (69.0)**
Not indicated	39 (16.4)*	18 (24.0)**
Missing	42 (17.6)*	4 (5.3)**

*% calculated from all patients admitted to the hospital (n = 238)

**% calculated from Utstein subgroup patients admitted to the hospital (n = 75)

**Table 2:** EMS times

Time period	Total/All cases		Utstein group	
	n	Median (IQR)	n	Median (IQR)
Departure to scene	738	9 (7–11)	132	8 (7–10)
Departure to patient	681	10 (8–12)	132	9 (8–11)
Departure to defibrillation	242	11 (9–14)	133	11 (9–13)
Departure to epinephrine	660	15 (12–18)	111	15 (12–17)
Scene to emergency department	248	6 (5–9)	78	6.5 (5–9)

All values reported in minutes

### Outcome measures

The primary outcome measure of this study was survival to hospital discharge in all patients and in the Utstein comparator subgroup which is defined as bystander-witnessed OHCA of medical/cardiac aetiology with an initial shockable rhythm. We selected these two groups because they reflect EMS system effectiveness and efficacy, respectively, according to the Utstein template [3]. 1-year survival was measured as a secondary outcome.

### Statistical analysis

Age and EMS times were reported as the medians and interquartile ranges (IQRs). To compare the times of occurrence, the Mann-Whitney U and Wilcoxon tests were used. Categorical variables are reported as numbers and proportions and were compared by using the Pearson chi-square test. To assess the associations among patient (age, sex), arrest (location, first rhythm) and care (bystander CPR, defibrillation) characteristics and the odds of being alive at hospital discharge, logistic regression models were used. The log linearity was tested for continuous variables, and variables for which log linearity was not proven were converted into categorical variables. First, a univariate analysis was performed with all the descriptive variables, and the association between these variables and the survival rate was tested using the Wald test. Stratification was then performed on OHCA characteristics with adjustment for age and sex. Multivariate analysis was performed with adjustment for all the variables that were significant in the univariate analysis. To assess the impact of patient characteristics, odds ratios (ORs) and their 95% confidence intervals (95% CIs) and p-values are presented.

All tests were two-tailed, and p-values less than 0.05 were considered to indicate statistical significance.

## Results

**Fig. 1:**
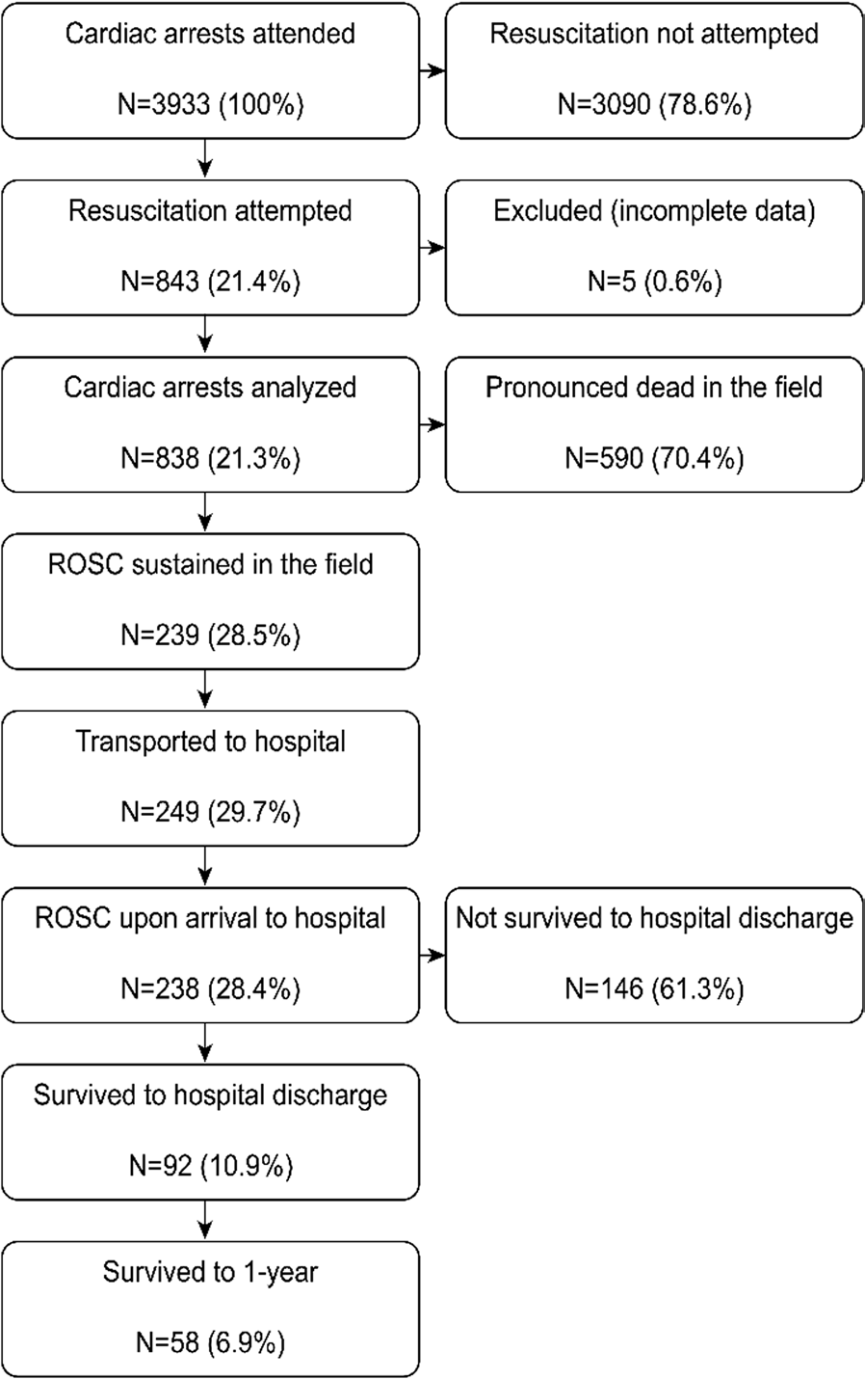
Study flowchart

In total, 838 OHCA cases of EMS-treated CA were reported (95.8 cases/100.000 inhabitants/year). A flowchart of the study participants is presented in Fig. 1. EMS-treated OHCA cases constituted 21.4% of all EMS-attended cases. Medical dispatchers identified OHCA in 50.0% of all cases and provided over-the-phone CPR instructions in 44.6% of them. Approximately 19% of OHCA patients were still alive during the call. Approximately 19% of all dispatcher data were missing because data were not saved to the EMS database. The median EMS response time (from departure to arrival at the patient) was 10 min. Other EMS times are presented in Table 2.

### Patient characteristics

Detailed characteristics of all study participants and the Utstein comparator subgroup are presented in Table 1. The median age was 71 (IQR 58–81) years, and 66.7% of patients were males. A total of 73.8% of OHCA cases occurred at home, 59.3% were witnessed by a bystander, and 54.5% received bystander cardiopulmonary resuscitation (CPR). A presumed medical aetiology was reported in 78.8% of all cases. One patient received a shock from an AED before EMS arrival. The initial rhythm was shockable in 27.6% and nonshockable in 68.5% of all cases.

### Outcomes

Return of spontaneous circulation (ROSC) at hospital transfer was evident in 24.9% of all cases. The survival to hospital discharge rate was 10.9%, and the 1-year survival rate was 6.9%. The survival to hospital discharge rate in the Utstein comparator group was 47.1%, and the 1-year survival rate was 27.5%. Patient outcomes were reported in accordance with the Utstein recommendations and presented in Table 3.

**Table 3:** Patient outcomes reported in accordance with the Utstein recommendations

Patient outcomes reported	ROSC sustained to hospital		Survived to hospital discharge		1-year survival	
	Yes N (%)	No N (%)	Yes N (%)	No N (%)	Yes N (%)	No N (%)
*All EMS-treated arrests*						
2016–2018 n = 838	238 (28.4)	600 (71.6)	92 (10.9)	746 (89.0)	58 (6.9)	780 (93.1)
2016 n = 312	84 (26.9)	228 (73.1)	27 (8.7)	285 (91.3)	12 (3.8)	300 (96.2)
2017 n = 281	79 (28.1)	202 (71.9)	34 (12.1)	247 (87.9)	22 (7.8)	259 (92.2)
2018 n = 245	75 (30.6)	170 (69.4)	31 (12.7)	214 (87.3)	24 (9.8)	221 (90.2)
*Shockable bystander-witnessed (EMS-witnessed excluded)*						
2016–2018 n = 138	75 (54.3)	63 (45.7)	65 (47.1)	73 (52.9)	38 (27.5)	100 (72.5)
2016 n = 42	21 (50)	21 (50)	13 (31)	29 (69)	9 (21.4)	33 (78.6)
2017 n = 49	27 (55.1)	22 (44.9)	26 (53.1)	23 (46.9)	11 (22.4)	38 (77.6)
2018 n = 47	27 (57.4)	19 (40.4)	26 (55.3)	21 (44.7)	18 (38.3)	29 (61.7)
*Shockable bystander CPR (EMS-witnessed excluded) N = 165*						
2016–2018	83 (50.3)	82 (49.7)	56 (33.9)	109 (66.1)	38 (23.0)	127 (77)
2016 n = 58	23 (39.7)	35 (60.3)	14 (24.1)	44 (75.9)	5 (8.6)	53 (91.4)
2017 n = 50	28 (56.0)	22 (44)	18 (36)	32 (64)	12 (24.0)	38 (76)
2018 n = 57	32 (56.1)	25 (43.9)	24 (42.1)	33 (57.9)	21 (36.8)	36 (63.2)
*Non-shockable bystander-witnessed (EMS witnessed excluded)*						
2016–2018 n = 318	61 (19.2)	257 (80.8)	7 (2.2)	311 (97.8)	3 (0.9)	315 (99.1)
2016 n = 108	23 (21.3)	85 (78.7)	2 (1.9)	106 (98.1)	0 (0.0)	108 (100)
2017 n = 132	23 (17.4)	109 (82.6)	5 (3.8)	127 (96.2)	3 (2.3)	129 (97.7)
2018 n = 78	15 (19.2)	62 (79.5)	0 (0.0)	78 (100)	0 (0.0)	78 (100)

None of the discharged OHCA patients were evaluated using the Cerebral Performance Category (CPC) or modified Rankin Scale (mRS). 48% of all surviving patients were discharged to rehabilitation facilities, 21.7% to other hospitals, 16.3% to home, and 12% to nursing facilities.

### Factors associated with improved survival to hospital discharge

In the univariate model, five statistically significant factors were associated with improved survival to hospital discharge: shockable rhythm, time from call to arrival at the patient less than 10 min, witnessed OHCA, age < 80 years, and male sex. The multivariate model showed a statistically significant effect of shockable rhythm (OR 14.55, 95% CI 7.35–28.82), time from call to arrival at the patient less than 10 min (OR 2.18 95% CI 1.19–4.01), and age < 80 years (OR 2.96, 95% CI 1.18–7.41). Witnessed OHCA (OR 1.95, 95% CI 0.97–3.64) and male sex (OR 1.44, 95% CI 0.70–2.97) were statistically nonsignificant in the multivariate model (Table 4).

**Table 4:** Multivariate logistic regression analysis for survival to hospital discharge in all EMS-treated OHCA cases

Variable	OR	95 CI	*p*
Male sex	1.44	0.70–2.97	0.325
Age ≤ 80	2.96	1.18–7.41	0.020
Shockable rhythm	14.55	7.35–28.82	< 0.001
Witnessed arrest	1.95	0.97–3.64	0.063
EMS departure to scene ≤ 10 min	2.18	1.19–4.01	0.012

Odds ratios (ORs) and 95 confidence intervals from logistic regression are presented. Regressions controlled for sex.

## Discussion

This is the first study reporting epidemiology and outcomes of OHCA in Lithuania for a period of three years. The overall survival to hospital discharge rate was 10.9% in our study. Among the patients in the Utstein comparator group, the survival to hospital discharge rate was 47.1%. Analysis of Utstein comparator subgroup variables allows a better comparison with other systems. Our results are slightly better than those reported in the EuReCA TWO study (8% and 31%) [1] and in the Cardiac Arrest Registry to Enhance Survival (CARES) annual report in 2017 (10.4% and 32.6%, respectively) [4]. The overall 1-year survival rate in our study was 6.9%, and that for the Utstein group was 27.5%. The survivors in the Utstein comparator subgroup constituted 65.5% of the total number of 1-year survivors. A recent meta-analysis of 141 OHCA studies reported an overall 1-year survival rate of 7.7% [1].

The incidence of EMS-treated OHCA in Kaunas was 95.8 cases/100,000 inhabitants/year. This incidence is higher than that reported in the EuReCa TWO study (56 cases/100,000 inhabitants/year) [2], CARES (74.3 cases/100,000 inhabitants/year) [4], and AusROC Epistry (47.6 cases/100,000 inhabitants/year) in 2015 [5]. The OHCA incident rate in Kaunas was higher than that in other North European countries, such as Denmark [6], Finland [7], Sweden [8], Norway [9], and neighbouring Poland [10]. The proportion of cases in which resuscitation was initiated or continued by EMS staff was 21.4%, compared with 62.6% in the EuReCa TWO study [2]. The total population served by Kaunas EMS decreased from 0.36 million in 2007 to 0.29 million in 2017, but the number of EMS-treated CAs increased 5-fold from 19.96 cases/100,000 inhabitants/year in 2007 [11] to 97.4 cases/100,000 inhabitants/year in 2017. The increased number of CPR attempts could be related to the number of attempts performed in patients in whom CPR was regarded as futile earlier. The negative correlation between the frequency of CPR attempts and the incidence of shockable rhythm may confirm such an assumption [10]. In a study performed in Kaunas in 2007 [11], there were 43 CPR attempts per 100,000 persons per year, and the occurrence of ventricular fibrillation/pulseless ventricular tachycardia (VF/pVT) was equal to 48%. In our study the rate of shockable rhythm was 27.5%. The higher OHCA incidence rate in Kaunas is assumed to be because resuscitation is started more often because we do not have a do-not-resuscitate order in Lithuania.

BLS teams should start CPR in all OHCA cases, except when there are injuries incompatible with life or the presence of rigor mortis or lividity. Family members or other bystanders often expect CPR even in futile OHCA cases.

Our study revealed that 54.5% of OHCA patients received bystander CPR before EMS arrival. This is in accordance with EuReCa TWO data (58%) [12] but lower than the rate in other Northern European countries: Norway—80% [9], Denmark—80.6% [13] and Sweden—68.2% [14]. Lower bystander CPR rates could be related to a lack of national initiatives encouraging CPR training and application in public. There is no CPR training for schoolchildren or national AED program in Lithuania. The first responder programme, “AED ALERT”, was only implemented in 2019 and is only in the Kaunas region. In 2016, there were only 5 AEDs in public places in Kaunas with a link to the EMS. The AED network in Kaunas only started to grow in 2018. This is the main reason why only one person was successfully defibrillated with an AED before EMS arrival during the study.

In our study population, we found five factors related to survival to hospital discharge, three of which were statistically significant in the multivariate model: shockable rhythm, age ≤ 80 years, and EMS time to scene ≤ 10 min (Table 4). All of them are well known from other studies [15, 16]. A study from 12 OHCA registries found that an initial shockable rhythm had the strongest association with survival to hospital discharge, and increasing patient age and time to EMS assessment were consistently associated with poorer survival. In addition, public location, witnessed events and bystander defibrillation were consistently associated with improved odds of survival [15]. Al-Dury et al. [16] also found that the most important predictor of survival in OHCA is the initial rhythm, followed by age, time to start of CPR, EMS response time and place of OHCA.

Our study has revealed some systemic flaws in OHCA care. Only 2.6% of OHCA patients received targeted temperature management in the hospital. In contrast, CARE reported that 45.5% of admitted patients received TTM [4]. There was no standard post-resuscitation care protocol (including the use of TTM), even in hospitals with 24/7 percutaneous cardiac intervention (PCI) facilities until recently.

We were also not able to report neurologic outcomes according to the Utstein recommendations because none of the discharged OHCA patients were evaluated using the CPC or mRS. We presumed that patients discharged to rehabilitation facilities (48.9%) had favourable neurologic outcomes and patients discharged to nursing facilities (most of them with tracheostomies, remaining in a state of coma) had unfavourable neurologic outcomes (12%).

Our study had several limitations, which appeared only at the statistical analysis stage. We found a lack of information about mechanical chest compression device usage, which is probably associated with the EMS database entry process. AED usage before ambulance arrival is not clear for the same reason as stated for mechanical chest compression device use. Regarding technical difficulties, we lacked some dispatch centre call recordings for the period from May to December 2018.

Our study provides a baseline for future reference, summarizing patient characteristics, processes and outcomes for OHCA.

## Conclusion

This study is a small step towards a national, population-based CA registry that would allow systematic assessment of the strengths and weaknesses of the chain of survival in Lithuania.

Routine OHCA data collection and analysis will allow us to track the efficacy of service improvements and should become a standard practice in all Lithuanian regions.

## Data Availability

The datasets used and/or analysed during the current study are available from the corresponding author on reasonable request.

## Abbreviations

AED: Automated external defibrillator
ALS: Advanced life support
BLS: Basic life support
CA: Cardiac arrest
CI: Confidence interval
CPC: cerebral performance category
CPR: Cardiopulmonary resuscitation
DNAR: Do not attempt resuscitation
EMS: Emergency medical service
ERC: European Resuscitation Council
IQR: Interquartile ranges
MPDS: Medical Priority Dispatch System
mRS: Modified Rankin Scale
OHCA: Out-of-hospital cardiac arrest
OR: Odds ratio
PCI: Percutaneous coronary intervention
PEA: Pulseless electrical activity
ROSC: Return of spontaneous circulation
TTM: Targeted temperature management
VF: Ventricular fibrillation
VT: Ventricular tachycardia
